# When Mind and Body Collide: Psychiatric and Orthopedic Challenges in a Clinical Case

**DOI:** 10.1192/j.eurpsy.2026.11666

**Published:** 2026-07-31

**Authors:** I. M. Peso Navarro, L. Garcia Avellaneda, C. Payo Rodriguez, R. A. Soto Limo, C. Marin Lorenzo, N. M. Casado Espada

**Affiliations:** 1 Psiquiatría, Complejo Asistencial Universitario de Salamanca, Salamanca; 2 Traumatología, Hospital General de Merida, Merida, Spain

## Abstract

**Introduction:**

The interaction between psychiatric disorders and orthopedic injuries is clinically relevant but often underestimated. Self-harming behaviors, impulsivity, and impaired judgment in psychiatric patients may result in traumatic injuries requiring specialized medical care. These cases demand close collaboration between psychiatry and traumatology to optimize outcomes.

**Objectives:**

To present a clinical case illustrating the overlap between psychiatric symptoms and traumatic injury.

To emphasize the importance of an integrated, multidisciplinary approach.

To discuss diagnostic and therapeutic challenges.

**Methods:**

We report the case of an adolescent patient admitted to the emergency department following a fracture sustained during an impulsive act related to psychiatric symptomatology. Data were collected from emergency records, psychiatric evaluations, and follow-up in both services.

**Results:**

The patient, with a history of mood instability and self-harm, presented with a distal radius fracture following an episode of agitation and impulsivity.

- Traumatology: Urgent orthopedic stabilization was performed, followed by routine postoperative care.
- Psychiatry: Comprehensive assessment revealed underlying depressive symptoms and emotional dysregulation. Psychotherapy and pharmacological treatment with SSRIs were initiated.
- Multidisciplinary care: Joint follow-up visits allowed coordination of physical rehabilitation and psychiatric stabilization.
  After several months, fracture healing was achieved, and the patient showed significant improvement in mood regulation and reduction of self-harming behaviors.

**Conclusions:**

This case highlights the critical intersection of psychiatry and traumatology. Traumatic injuries in psychiatric patients are not merely physical events but markers of psychological vulnerability. A collaborative, multidisciplinary approach ensures both physical recovery and mental health stabilization, ultimately improving prognosis.

**Disclosure of Interest:**

None Declared

